# rs71327024 Associated with COVID-19 Hospitalization Reduces *CXCR6* Promoter Activity in Human CD4^+^ T Cells via Disruption of c-Myb Binding

**DOI:** 10.3390/ijms241813790

**Published:** 2023-09-07

**Authors:** Aksinya N. Uvarova, Ekaterina M. Stasevich, Alina S. Ustiugova, Nikita A. Mitkin, Elina A. Zheremyan, Savely A. Sheetikov, Ksenia V. Zornikova, Apollinariya V. Bogolyubova, Mikhail A. Rubtsov, Ivan V. Kulakovskiy, Dmitry V. Kuprash, Kirill V. Korneev, Anton M. Schwartz

**Affiliations:** 1 Center for Precision Genome Editing and Genetic Technologies for Biomedicine, Engelhardt Institute of Molecular Biology, Russian Academy of Sciences, 119991 Moscow, Russia; ogstasevich@gmail.com (E.M.S.); ustugovaalina@yandex.ru (A.S.U.); mitkin.n.a@gmail.com (N.A.M.); elyazheremyan@mail.ru (E.A.Z.); kuprash@gmail.com (D.V.K.); 2 Faculty of Biology, Lomonosov Moscow State University, 119234 Moscow, Russia; rijik99@gmail.com (S.A.S.); zornikovaksenia@yandex.ru (K.V.Z.); ma_rubtsov@mail.ru (M.A.R.); 3 National Research Center for Hematology, 125167 Moscow, Russia; apollinariya.bogolyubova@gmail.com; 4 Institute of Protein Research, 142290 Pushchino, Russia; ivan.kulakovskiy@gmail.com; 5 Department of Human Biology, Faculty of Natural Sciences, University of Haifa, 199 Abba Khoushy Avenue, Mount Carmel, Haifa 3498838, Israel; aschwart2@univ.haifa.ac.il

**Keywords:** 3p21.31 locus, COVID-19, CXCR6, T helpers, c-Myb, non-coding SNP

## Abstract

Single-nucleotide polymorphism rs71327024 located in the human 3p21.31 locus has been associated with an elevated risk of hospitalization upon SARS-CoV-2 infection. The 3p21.31 locus contains several genes encoding chemokine receptors potentially relevant to severe COVID-19. In particular, CXCR6, which is prominently expressed in T lymphocytes, NK, and NKT cells, has been shown to be involved in the recruitment of immune cells to non-lymphoid organs in chronic inflammatory and respiratory diseases. In COVID-19, CXCR6 expression is reduced in lung resident memory T cells from patients with severe disease as compared to the control cohort with moderate symptoms. We demonstrate here that rs71327024 is located within an active enhancer that augments the activity of the *CXCR6* promoter in human CD4^+^ T lymphocytes. The common rs71327024(G) variant makes a functional binding site for the c-Myb transcription factor, while the risk rs71327024(T) variant disrupts c-Myb binding and reduces the enhancer activity. Concordantly, c-Myb knockdown in PMA-treated Jurkat cells negates rs71327024’s allele-specific effect on *CXCR6* promoter activity. We conclude that a disrupted c-Myb binding site may decrease CXCR6 expression in T helper cells of individuals carrying the minor rs71327024(T) allele and thus may promote the progression of severe COVID-19 and other inflammatory pathologies.

## 1. Introduction

Genome-wide association studies reported multiple associations between single-nucleotide polymorphisms (SNPs) and COVID-19 outcome and severity [1]. Among multiple non-coding SNPs involved, several SNPs in the 3p21.31 locus were associated with severe COVID-19, including rs13078854, rs35081325, rs2271616, rs10490770, and rs11385942 [2,3,4]. The 3p21.31 locus contains putative SARS-CoV-2 coreceptors (*SCL6A20*, *LZTFL1*, and *FYCO1*) as well as several chemokine receptors (*CXCR6*, *XCR1*, *CCR1*, *CCR2*, *CCR3*, *CCR5*, and *CCR9*), whose involvement in COVID-19 infection has been recently suggested [5,6,7,8,9].

Chemokines and their receptors are crucial for the recruitment of effector immunocytes to the site of inflammation, a critical stage in the effective response to respiratory pathogens. However, in COVID-19, modulation of chemokine response can be a direct cause of acute respiratory disease syndrome, a serious complication leading to death in approximately 40% of severe cases [10]. Likewise, CXCR6 may be involved in tumor progression [11] and participate in the recruitment of immune cells to non-lymphoid organs during chronic inflammation [12,13] and acute respiratory diseases [14,15]. This receptor is expressed on a variety of lymphocytes such as NK cells, NKT cells, naive CD8^+^ T cells, activated CD4^+^ and CD8^+^ T cells, as well as γδT cells [11]. CXCR6 acts as a receptor for the pro-inflammatory chemokine CXCL16, which is highly expressed in the lungs, spleen, and whole blood (https://www.gtexportal.org/home/gene/CXCL16, accessed on 1 August 2022) [16].

Recent studies revealed that the CXCR6/CXCL16 axis plays an important role in severe COVID-19 immunopathogenesis [17]. CXCR6 expression by lung-resident memory CD8^+^ T cells was lower in patients with severe COVID-19, indicating the possible protective role of CXCR6^+^ killer T cells [18,19]. In addition, according to single-nucleus RNA-seq data, CXCR6 expression was dysregulated in lung samples from the cohort of patients who have died from COVID-19, with marked downregulation in some of the samples [20]. COVID-19 is characterized by a decreased number of CD8^+^ and CD4^+^ T cells in the blood and in lung tissue [21,22], and the severity of the disease is directly related to the lymphocytopenia grade [23]. The decreased CD4^+^ T cell level is even used as an independent predictor for in-hospital death in COVID-19 patients [24]. CD4^+^ T cells function during acute SARS-CoV-2 infection is an intriguing, widely discussed question that is left to be answered [25].

A set of studies of eQTLs (expression quantitative trait loci) reveals that the COVID-19 severity may be mediated by genetic variants associated with CXCR6 expression [19]. In this study, we focused on SNP rs71327024, which is an eQTL for the *CXCR6* gene. This SNP is associated with COVID-19-related hospitalization [26] and is located in a non-coding region with the epigenetic marks of an active regulatory element in the human 3p21.31 locus [27]. We studied the effect of the minor rs71327024(T) allele on the activity of the human *CXCR6* promoter in T helpers using a reporter assay. We showed that impaired c-Myb binding at the rs71327024(T) allele results in reduced *CXCR6* transcription, providing a possible explanation for the harmful effect of this single-nucleotide variant in the context of COVID-19 pathogenesis.

## 2. Results

### 2.1. The Risk Allele (T) of rs71327024 Decreases the CXCR6 Promoter Activity in Jurkat, CEM and CD4^+^ Primary Cells

According to the data of the COVID-19 Host Genetics Initiative [26], rs71327024 is associated with COVID-19-related hospitalization (*p* < 10^−4^). This SNP is located in the 3p21.31 locus and is an eQTL for multiple immune response genes (predominantly chemokine receptors) in several tissues (according to GTEx) [16]. The minor allele frequency of rs71327024 is heterogeneous among the global population: the risk T allele is most common in South Asian and less represented among African (Supplementary Table S1). According to ENCODE [28] and Roadmap epigenomic data [29] visualized by the UCSC Genome Browser (http://genome.ucsc.edu/, accessed on 1 February 2022), the genomic region surrounding rs71327024 has epigenetic marks of enhancer regions (chr3:46097185-46099619; GRCh38/hg38): high level of H3K4me1 and H3K27ac histone modifications [27], DNase I hypersensitivity sites and clusters of transcription factor binding sites (Figure S1).

Since rs71327024 is an eQTL for the *CXCR6* gene that has several RNA splice variants (Figure S1), we had to choose an isoform expressed predominantly in T helpers, well known players in COVID-19 pathogenesis [25]. For functional validation of promoter activity in CD4^+^ cells, we cloned putative *CXCR6* promoter regions (chr3:45942118-45946481, promoter 1; chr3:45939898-45941054, promoter 2; all GRCh38/hg38) (Figure S2a) into a pGL3-basic reporter vector. Using a luciferase reporter assay, we tested the activity of constructs in an experimental model of T helper cells, the PMA-treated human T-lymphoblastoid Jurkat cell line [30,31,32]. Promoter 1, located in the active chromatin region, showed high activity, while promoter 2 was significantly less active (Figure S2b). These results can be connected with the location of the cell-type specific TSSs (transcription start sites), according to data from FANTOM5 hg38 human promoterome collection [33]. Promoter 1 contained TSSs with the highest CAGE (Cap Analysis of Gene Expression) tag counts for CD4^+^ cells (ENST00000304552.5, ENST00000458629.1, and ENST00000457814.1 isoforms), whereas promoter 2, located upstream of the isoform ENST00000438735.1, includes a TSS that predominantly controls isoform expression in chorionic membrane cells (Figure S2a). Thus, for further experiments, we selected promoter 1, which is common for major CD4^+^ cell-specific *CXCR6* isoforms.

**Figure 1:**
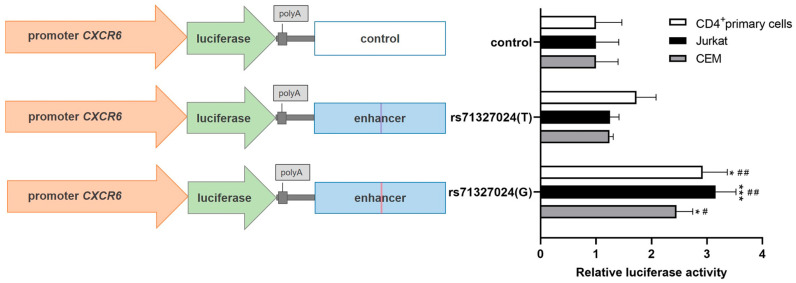
3p21.31 locus enhancer containing common rs71327024(G) allele but not the rare (T) variant significantly increased *CXCR6* promoter activity in PMA-treated Jurkat, CEM and primary CD4^+^ T cells. Left: Enhancer region of 3p21.31 locus containing common (G) or minor (T) alleles of rs71327024 or irrelevant control fragment was cloned downstream of the luciferase gene placed under the *CXCR6* promoter in the pGL3-basic vector. Right: Relative luciferase activity in Jurkat, CEM and CD4^+^ primary cells transfected with constructs containing *CXCR6* promoter and enhancer of the 3p21.31 locus with various rs71327024 alleles or control sequence. All data were normalized to Renilla luciferase internal reference and further normalized to the luciferase activity of controls in CD4^+^ primary or Jurkat or CEM cells. Data represent eleven (Jurkat and CD4^+^ primary cells) and three (CEM cells) independent experiments with mean values ± SEM. * *p* < 0.05, *** *p* < 0.001 compared to the rs71327024(T) allele, # *p* < 0.05, ## *p* < 0.01 compared to the control, as calculated by unpaired Student’s *t*-test.

The reporter plasmid with the putative enhancer containing the common rs71327024(G) allele demonstrated a 2.5-fold increase in luciferase activity compared to the vector containing an irrelevant sequence in all activated Jurkat, CEM and primary CD4^+^ cells (Figure 1). Conversion of the rs71327024(G) variant to the minor T allele abrogated the enhancer activity, suggesting that the selected region of the 3p21.31 locus acts as an enhancer of the *CXCR6* promoter in T helpers only in the presence of the common rs71327024(G) allele. 

### 2.2. The Common rs71327024(G) Makes a c-Myb Binding Site in PMA-Treated Jurkat Cells

The ADASTRA database [34] reports that rs71327024 serves as a direct allele-specific binding site of the transcription factor c-Myb (highlighted in [35]) in Th1 cells. c-Myb is one of the key regulators of mammalian hematopoiesis and is involved in the regulation of fetal hemoglobin expression and the maturation of T and B lymphocytes [36]. Moreover, c-Myb appears to be important for the differentiation and maintenance of the Th2 phenotype by CD4^+^ T cells [37], and c-Myb expression is increased in PMA-treated Jurkat cells [38].

**Figure 2:**
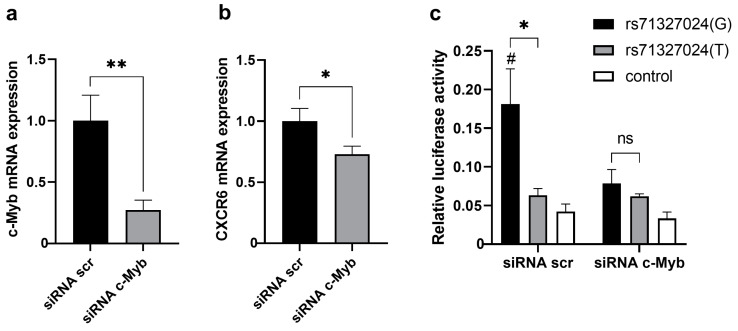
c-Myb knockdown removes the rs71327024 allele-dependent difference in activity of enhancer at the 3p21.31 locus and downregulates *CXCR6* expression in PMA-treated Jurkat cells. Relative expression levels of c-Myb (**a**) and *CXCR6* (**b**) mRNA in PMA-treated Jurkat cells transfected with anti-c-Myb siRNA and control scrambled siRNA (siRNA scr) according to qRT-PCR data. The data normalized to β-actin and further normalized to the expression levels mRNA with control scrambled siRNA. Data represent at least five independent experiments with mean values ± SEM. ** *p* < 0.01, * *p* < 0.05, as calculated by unpaired Student’s *t*-test. (**c**) Relative luciferase activity in cells transfected with constructs containing *CXCR6* promoter and enhancer at 3p21.31 locus with alternative rs71327024 alleles or control irrelevant sequence and also transfected with anti-c-Myb siRNA and control scrambled siRNA (siRNA scr). All data were normalized to Renilla luciferase internal reference. Data represent four independent experiments with mean values ± SEM. * *p* < 0.05, as calculated by unpaired Student’s *t*-test, # *p* < 0.05, unpaired Student’s *t*-test compared to the control, ns, not significant.

To test the hypothesis that the regulatory effect of rs71327024 on *CXCR6* expression is mediated through altering c-Myb binding, we performed siRNA-mediated knockdown of c-Myb in PMA-treated Jurkat cells. c-Myb expression was suppressed almost 4-fold (Figure 2a) and resulted in a slight downregulation of *CXCR6* (Figure 2b). Knockdown of c-Myb led to the equalization of the relative activity of the enhancers carrying alternative rs71327024 alleles in PMA-treated Jurkat cells (Figure 2c). 

To validate the allele-specific c-Myb binding to rs71327024, amplicons containing the enhancer sequence with alternative variants of rs71327024 were mixed with nuclear extracts of PMA-treated Jurkat cells and were immunoprecipitated with antibodies against c-Myb (DNA pull-down assay). This method includes immunoprecipitation of DNA–protein complexes in vitro with subsequent quantitation of the bound amplicons by real-time PCR (Figure S3).

**Figure 3:**
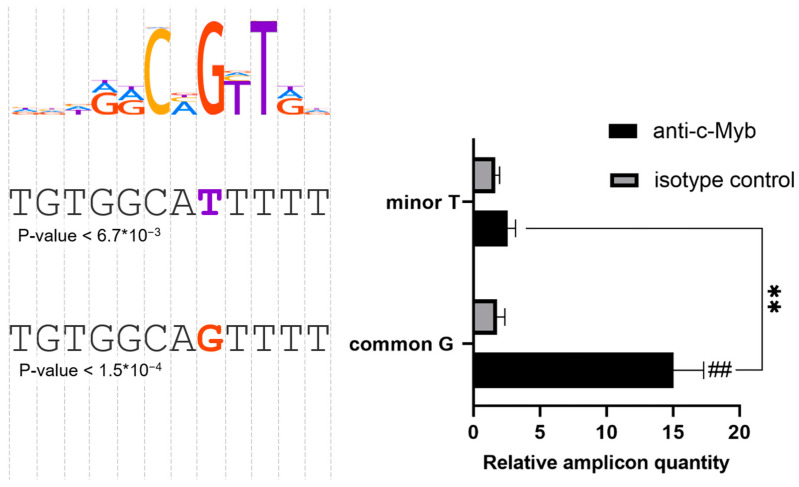
The common allele of rs71327024(T) of enhancer at 3p21.31 locus disrupts the c-Myb binding site in PMA-treated Jurkat cells. Left: The c-Myb motif logo from HOCOMOCO v11 database aligned with the corresponding segments of the enhancer sequence with different alleles SNP rs71327024. The motif *p*-values are indicated below the sequences. Right: Pull-down assay with anti-c-Myb antibodies and nuclear extract from PMA-treated Jurkat cells. The amplicon amount was quantified by real-time PCR and normalized to the value obtained with the negative control DNA fragment. The presented data represents three independent experiments with mean values ± SEM. ** *p* < 0.01, as calculated by unpaired Student’s *t*-test. ## *p* < 0.01 as calculated by unpaired Student’s *t*-test compared to the isotype control.

The DNA amplicon with the common rs71327024(G) allele was expected to provide a robust c-Myb binding site compared to the risk rs71327024(T) allele (Figure 3, left). Indeed, the probe carrying the common rs71327024(G) variant bound c-Myb approximately 15 times more efficiently than the amplicon with the risk rs71327024(T) allele (Figure 3, right). Thus, our data indicate that the activity of the 3p21.31 locus enhancer with the common rs71327024(G) allele in T-helper cells depends on the active c-Myb binding site. Introduction of the risk rs71327024(T) allele completely disrupts c-Myb binding. 

## 3. Discussion

**Figure 4:**
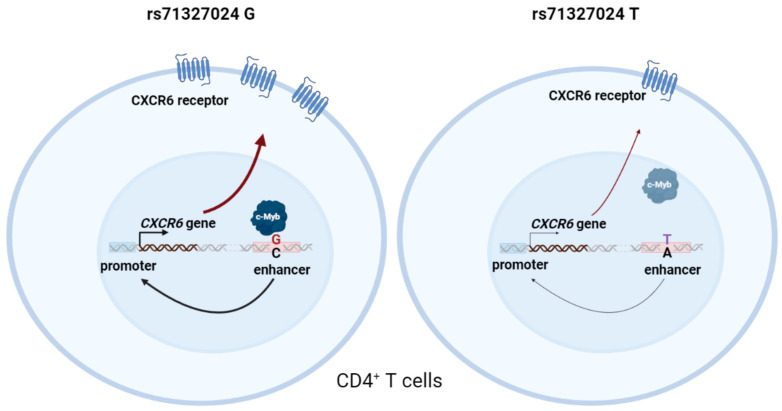
Transcription factor c-Myb binds to the genomic region surrounding the common rs71327024(G) allele, while the risk rs71327024(T) allele associated with COVID-19 hospitalization prevents c-Myb binding that lead to loss of enhancer properties and reduced *CXCR6* gene expression (created with BioRender.com).

The rs71327024 SNP is associated with COVID-19-related hospitalization and is located at the locus of immunologically relevant genes, including chemokine receptors. The functional potential of rs71327024 during COVID-19 progression has also been noted in other studies, primarily in macrophages and monocytes [39,40]. Our data suggest a possible mechanistic link explaining the association of the minor rs71327024(T) allele with COVID-19 severity through reduced CXCR6 expression, which is observed in T cells of SARS-CoV2 infected patients. We identified an enhancer region (chr3:46097189-46099620; GRCh38/hg38) carrying the rs71327024 polymorphism that normally increases *CXCR6* promoter activity in CD4^+^ T cells but loses its properties in the case of the risk allele rs71327024(T). We also showed that the binding of transcription factor c-Myb to the rs71327024 region is allele-specific, concordant with the results of siRNA-knockdown of c-Myb, which abolished the difference in enhancer activity between rs71327024(G) and rs71327024(T) in PMA-treated Jurkat cells. Thus, c-Myb binds to the rs71327024(G) while G to T substitution prevents c-Myb binding and leads to loss of enhancer properties and reduced CXCR6 expression (Figure 4).

As noted, despite the fact that the number of T helper cells during COVID-19 decreases in the blood and lungs, and their reduction is a marker of in-hospital mortality amongst patients, their function in the acute phase of infection is predominantly unclear [21,24]. In patients with respiratory diseases, the CXCR6/CXCL16 axis is known to attract T cells to the lungs [41] and this mechanism appears to play a role in the COVID-19 immunopathogenesis as well [42,43]. The number of CD4^+^ CXCR6^+^ T cells was reduced in the blood of patients with severe COVID-19 compared to those with a negative viral load [17]. It is possible that even a partial decrease in CXCR6 expression in T cells may have a significant effect on their trafficking during reduced CXCL16 production. Moreover, patients with severe COVID-19 have fewer lung-resident memory CD8^+^ T cells with lower expression of CXCR6, and their lung macrophages produced less CXCL16 compared to moderately ill patients [18,19]. Taken together, dysregulation of T lymphocyte migration to the lungs due to reduced expression of CXCR6 may provide an explanation for the exacerbated condition of COVID-19 patients harboring the risk rs71327024(T) variant.

In the present work, we have shown that the disrupted c-Myb binding site in the 3p21.31 locus may decrease CXCR6 expression in T helper cells of individuals carrying the risk rs71327024(T) allele and promote the progression of severe COVID-19. Methods used in our study present apparent limitations. In particular, reporter plasmid assays and in vitro DNA binding possibly lack proper chromatin context. In addition, siRNA-mediated c-Myb knockdown may bring about global cellular changes that would affect the regulation of the *CXCR6* gene indirectly. Further analysis of the potential regulatory properties of rs71327024 may be required to uncover the underlying molecular mechanisms. In order to eliminate some of the limitations mentioned above, seamless genome editing may be employed.

## 4. Materials and Methods

### 4.1. Cell Cultures

CD4^+^ primary T cells and the T lymphoblastoid Jurkat cell line (STR genotyping from May 26, 2022) were cultivated in RPMI 1640 medium (PanEco, Moscow, Russia) supplemented with 10% FBS (Corning, NY, USA), 2 mM L-glutamine, 1mM sodium pyruvate, 100 U/mL penicillin, and 100 μg/mL streptomycin (all PanEco, Moscow, Russia), non-essential amino acids, and 10 mM HEPES (all GIBCO, Kwartsweg, The Netherlands). CD4^+^ cells were isolated from peripheral blood mononuclear cells from healthy donors using a human CD4 MACS Cell Isolation Kit (Miltenyi Biotec, Bergisch Gladbach, Germany). Cell activation was performed by adding phorbol myristate acetate (PMA; Sigma-Aldrich, Burlington, MA, USA) at 50 ng/mL concentration to the culture medium the day before electroporation with test plasmids. All donors signed the informed consent form approved by the National Research Center for Hematology ethical committee before enrollment. 

### 4.2. Reporter Plasmids

To analyze *CXCR6* promoters activity we amplified the *CXCR6* putative promoter regions (chr3:45942118-45946481, promoter 1; chr3:45939898-45941054, promoter 2; all GRCh38/hg38) by PCR using human genomic DNA (Promega, Madison, WI, USA) as a template, and specific primers containing KpnI/XhoI restriction sites (Table S1). The promoter was cloned into a pGL3-basic luciferase reporter vector (Promega, Madison, WI, USA) using XhoI and KpnI restriction sites. We amplified the putative enhancer element, including rs71327024 (chr3:46,097,185-46,099,619), and a similarly sized irrelevant control sequence (an intergenic sequence without any epigenetic features of the regulatory region; chr3:83,749,648-83,752,164) by PCR using human genomic DNA (Promega, Madison, WI, USA) as a template, and specific primers containing BamHI/SalI restriction sites for enhancer and irrelevant control (Table S1). The putative enhancer element or control sequence was cloned into pGL3-basic vectors containing *CXCR6* promoter 1, using BamHI and SalI restriction sites downstream of the luciferase coding sequence and signal of polyadenylation. To analyze the influence of rs71327024(G/T), site-specific nucleotide changes in enhancer regions were introduced by two-stage PCR using internal overlapping primers (Table S1), as described previously [44]. Plasmid DNA was extracted and purified with the NucleoBond Xtra Midi Kit (Macherey-Nagel, Düren, Germany) and verified by Sanger sequencing (EIMB RAS “Genome” center, Moscow, Russia).

### 4.3. Cell Transfection and Luciferase Reporter Assay

For luciferase assay, 2.5 × 10^6^ cells were transfected with 5 μg of test plasmids combined with 0.5 μg of pRL-CMV Renilla luciferase control vector from the Dual-Luciferase Reporter Assay System (Promega, Madison, WI, USA). Cells were electroporated using the Neon Transfection System (Invitrogen, Carlsbad, CA, USA) with appropriate parameters: three 10 ms 1350 V pulses for Jurkat cells, one 20 ms 1400 V pulse for CEM cells, and two 15 ms 2000 V pulses for primary CD4+ cells. Luciferase activity was measured 24 h post-transfection using a 20/20n Luminometer (Turner BioSystems, Sunnyvale, CA, USA).

### 4.4. siRNA-Mediated c-Myb Knockdown

We used previously published short interfering RNA (siRNA) against the *MYB* gene [45]; appropriate sequences of the control scrambled RNA (scrRNA) were obtained by the InvivoGen siRNA Wizard v3.1 tool (Table S1). Single-stranded RNAs were commercially synthesized (“DNK-syntez”, Moscow, Russia) and annealed as previously described [46]. For c-Myb knockdown, 2.5 mln cells were transfected by electroporation (as described above) with 500 pmol c-Myb-specific siRNA or scrRNA duplexes. Cells were cultured for 48 h and then were transfected with 5 μg of test vectors, 0.5 μg of pRL-CMV control, and 300 pmol more of siRNA or scrRNA. Luciferase reporter assay was conducted after cell cultivation for 24 h.

### 4.5. DNA Pull-Down Assay

In order to assess c-Myb binding to DNA sequences containing different rs71327024 alleles, we performed a pull-down assay with PCR products based on the method described earlier [47]. The “Pull-down” primers (Table S1) were designed to produce 184 bp amplicons, corresponding to the sequences with minor and common rs71327024 variants; plasmids containing enhancer elements with different nucleotide variants were used as templates for the PCR reaction. Control amplicon was produced by PCR amplification with the “Pull-down-contr” primers pair (Table S1), including a 167 bp with no predicted c-Myb binding sites. All PCR products were verified by Sanger sequencing. Nuclear extracts were prepared from PMA-treated Jurkat cells using NE-PER Nuclear and Cytoplasmatic Extraction Reagents (Thermo Fisher Scientific, Rockford, IL, USA). DNA amplicons with rs71327024 T or G variant (100 ng), control DNA fragments (100 ng) and a nuclear extract (approximately 30 μg of total protein) were mixed in 100 μL of incubation buffer (60 mM KCl, 12 mM HEPES (pH 8.0), 4 mM TrisHCl (pH 8.0), 0.5 mM EDTA, 5% glycerol, 1 mM DTT) containing 5 μg Poly(dI-dC) sodium salt (Sigma-Aldrich, Burlington, MA, USA), and 1× protease inhibitor cocktail SIGMAFAST (Sigma-Aldrich, Burlington, MA, USA). After 1 h on ice, the sample was supplemented with 1 μg rabbit polyclonal IgG antibodies to c-Myb (ab226470, Abcam, Cambridge, MA, USA) or 1 μg rabbit polyclonal IgG anti-EBF1 (Antibodies-online Inc., Atlanta, GA, USA) as an isotypic control and was incubated one more hour on ice. Immunoprecipitation of DNA–protein complexes was performed using Protein A Mag Sepharose (GE Healthcare, Chalfont St Giles Buckinghamshire, UK). Then, 5 μL Protein A Sepharose beads were washed three times with buffer RIPA (50 mM TrisHCl, 150 mM NaCl, 2 mM EDTA, 1% NP-40, 0.5% Sodium Deoxycholate, 0.1% SDS); the beads were added to RIPA buffer containing competitor salmon sperm ssDNA (75 ng per 1 μL of beads), bovine serum albumin (0.1 μg per 1 μL of beads), and protease inhibitor cocktail SIGMAFAST (Sigma-Aldrich, Burlington, MA, USA). The mixture was incubated at room temperature for 30 min with continuous rotation. The beads were washed once with buffer RIPA and were diluted in RIPA to the original volume. The sample containing DNA fragments, nuclear extracts, and antibodies was combined with 5 μL of Protein A Mag Sepharose beads prepared as above. The suspension was incubated with overnight rotation at 4 °C. Then beads were washed using magnetic rack consecutively with Wash Buffer 1 (0.1% SDS, 1% Triton X-100, 2 mM EDTA, 20 mM Tris (pH 8.0), 150 mM NaCl), Wash Buffer 2 (0.1% SDS, 1% Triton X-100, 2 mM EDTA, 20 mM Tris (pH 8.0), 500 mM NaCl, and PBS. The DNA fragments were eluted with 20 μL of 2.5% acetic acid and neutralized with 7 μL of 10% NaHCO3. Eluted DNA was quantified by real-time PCR. 

### 4.6. RNA Extraction, cDNA Synthesis and Real-Time-PCR

Total RNA was isolated using the TRIzol reagent (Invitrogen, Carlsbad, CA, USA) as described in the manufacturer’s manual. Reverse transcription of total RNA was carried out using the MMLV RT Kit (Evrogen, Moscow, Russia) and mixed Oligo(dT) and random hexamers as primers. Real-time PCR analysis was performed using the CFX96 Touch real-time PCR detection system (Bio-Rad Laboratories, Hercules, CA, USA) and qPCRmix-HS SYBR (Evrogen, Moscow, Russia). β-Actin (*ACTB*) was used as a reference gene for *CXCR6* and *c-Myb* expression. The pull-down amplicon amount was normalized to the value obtained with the negative control DNA fragment. Sequences of oligonucleotide primers are presented in (Table S2).

### 4.7. Statistical Analysis

We performed statistical analyses using GraphPad Prism 9 software. Statistical significance was estimated using unpaired Student’s *t*-test. *p*-values < 0.05 were considered significant. Data for each sample represent the result of at least three independent experiments. Real-time PCR and luciferase assays were additionally performed in two technical replicates. All data are presented as mean ± SEM.

## Data Availability

The raw data supporting the results presented in this study are available upon request from the corresponding author.

## Supplementary Materials

The following supporting information can be downloaded at https://www.mdpi.com/article/10.3390/ijms241813790/s1.
