# Supplementary material for: rs71327024 Associated with COVID-19 Hospitalization Reduces CXCR6 Promoter Activity in Human CD4+ T Cells via Disruption of c-Myb Binding
Source: Int J Mol Sci. 2023 Sep 7;24(18):13790. doi: 10.3390/ijms241813790 (PMC10530726; doi:10.3390/ijms241813790)
Supplement: Supplementary file 1 [file ijms-24-13790-s001.zip › ijms-2527374-supplementary.pdf]

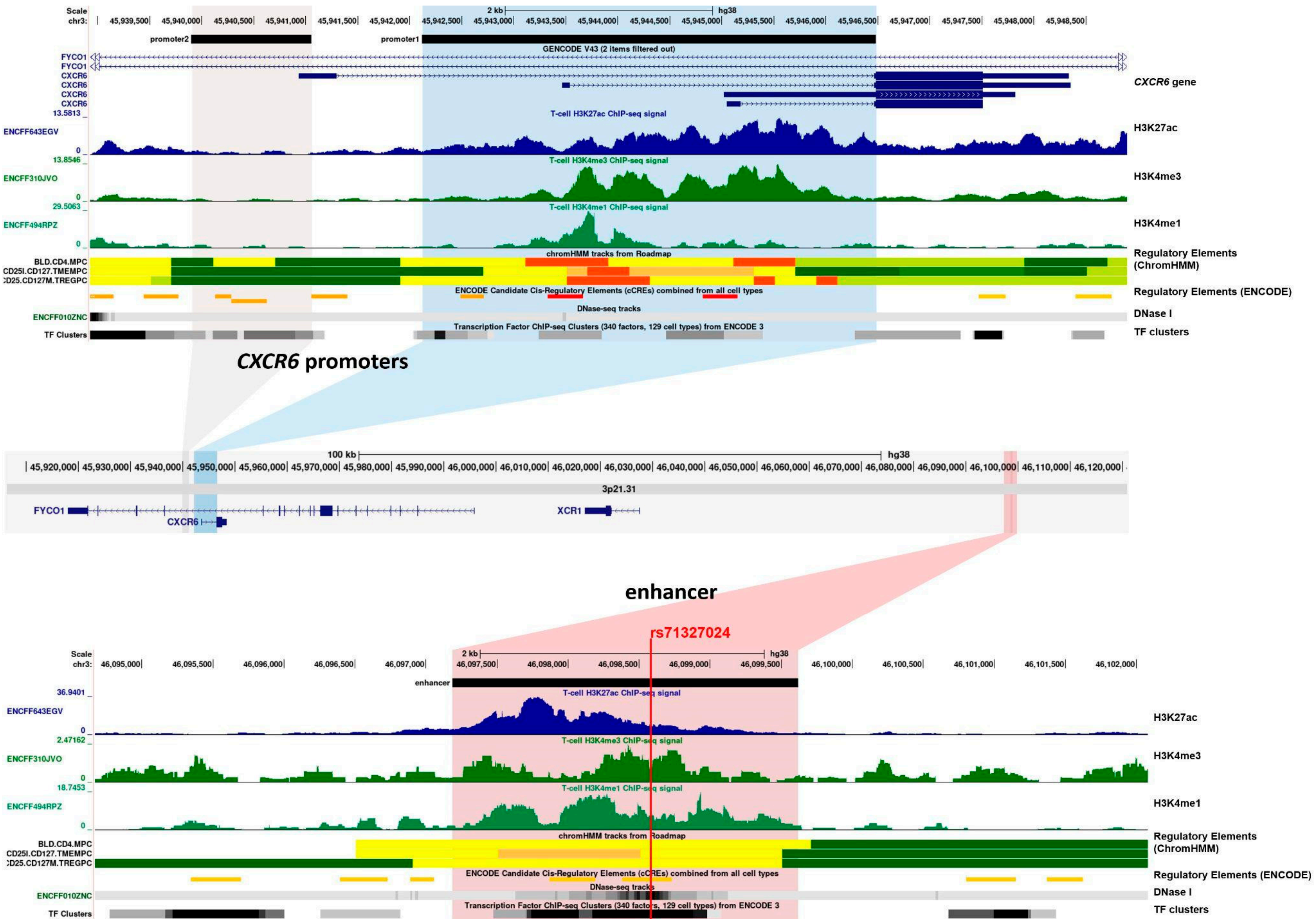

(legend is on the next page)

Figure S1. Schematic representation of the regulatory elements in the 3p21.31 locus around rs71327024 and *CXCR6* promoter based on the UCSC Genome Browser visualization (GRCh38/hg38). In the top section, light gray and light blue areas highlight the putative *CXCR6* promoter regions. In the bottom section, the light red area marks the putative enhancer in the 3p21.31 locus and the vertical red line shows the location of the rs71327024. Histograms indicate the location of histone modifications in T cells typical of regulatory elements (H3K4 mono/tri-methylation, H3K27 acetylation; Roadmap epigenomic data). Chromatin state segmentation in different subsets of T cells represents ChromHMM model (Roadmap): red and orange denote a state with a promoter-like signature, yellow denote states with an enhancer-like signature, green denotes a signature for transcribed region. Rectangles mark regulatory elements positions according to ENCODE, DNase I hypersensitivity clusters (DNase I) in T cells (Roadmap) and transcription factor ChIP-seq (TF clusters; ENCODE).

a

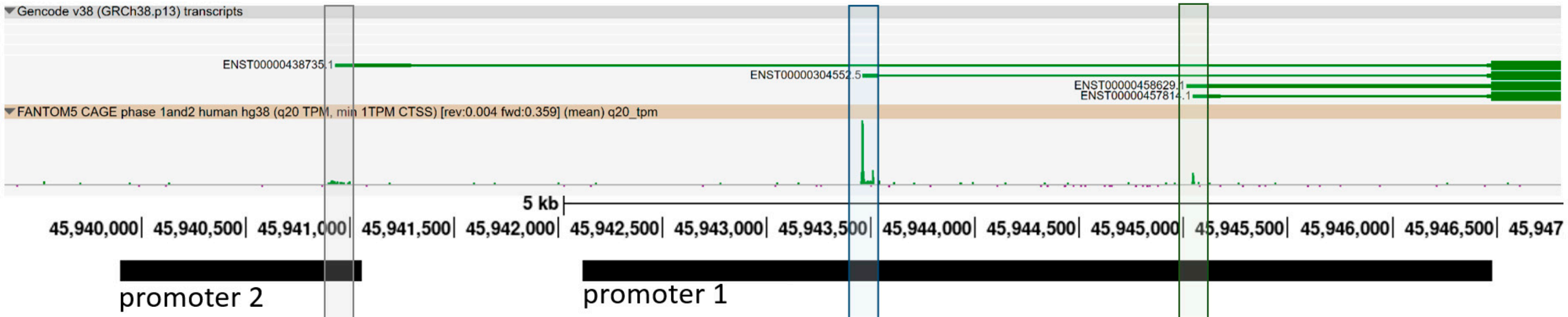

Cell type	CAGE tag counts
chorionic membrane cells, donor3	50.115
chorionic membrane cells, donor1	47.645
choriocarcinoma cell line:SCH	22.292
chorionic membrane cells, donor2	15.826
choriocarcinoma cell line:T3M-3	14.057
amniotic membrane cells, donor1	9.967
placenta, adult, pool1	9.273
pancreatic carcinoma cell line:NOR-P1	3.202
choriocarcinoma cell line:BeWo	2.495
Universal RNA - Human Normal Tissues	1.808

Cell type	CAGE tag counts
CD4+CD25-CD45RA- memory conventional T cells expanded, donor2	219.676
CD4+CD25-CD45RA- memory conventional T cells expanded, donor3	157.987
CD4+CD25+CD45RA- memory regulatory T cells expanded, donor1	144.358
gamma delta positive T cells, donor1	142.637
CD4+CD25-CD45RA+ naive conventional T cells expanded, donor2	138.766
CD4+CD25-CD45RA+ naive conventional T cells expanded, donor3	123.578
CD4+CD25-CD45RA- memory conventional T cells expanded, donor1	87.047
CD4+CD25+CD45RA- memory regulatory T cells expanded, donor3	80.933
CD4+CD25+CD45RA- memory regulatory T cells expanded, donor2	68.653
CD4+CD25-CD45RA+ naive conventional T cells expanded, donor1	64.083

Cell type	CAGE tag counts
CD4+CD25+CD45RA- memory regulatory T cells, donor1	17.723
gamma delta positive T cells, donor1	15.333
CD4+CD25-CD45RA- memory conventional T cells, donor1	12.087
CD8+ T Cells, donor1	11.455
CD4+CD25+CD45RA- memory regulatory T cells expanded, donor1	10.852
CD4+CD25+CD45RA- memory regulatory T cells, donor2	16316
Natural Killer Cells, donor1	7.435
CD4+CD25+CD45RA- memory regulatory T cells expanded, donor3	6.206
CD8+ T Cells (pluriselect), donor090325, donation2	5.667
CD8+ T Cells, donor2	5.586

b

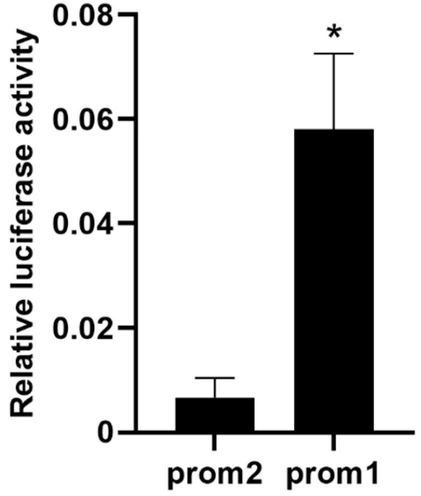

Figure S2. The activity of *CXCR6* promoter variants. (**a**) Schematic representation of the promoters and corresponding CAGE tag counts for three TSSs based on FANTOM5 hg38 human promoterome collection. Arrows highlight areas of TSSs and point to corresponding tables with specific CAGE tag counts for different cell types. (**b**) Relative luciferase activity in Jurkat cells transfected with constructs containing *CXCR6* promoter variants. All data were normalized to Renilla luciferase internal reference. Data represent five (promoter 1) and three (promoter 2) independent experiments with mean values ± SEM, * $p < 0.05$, as calculated by unpaired Student's t-test.

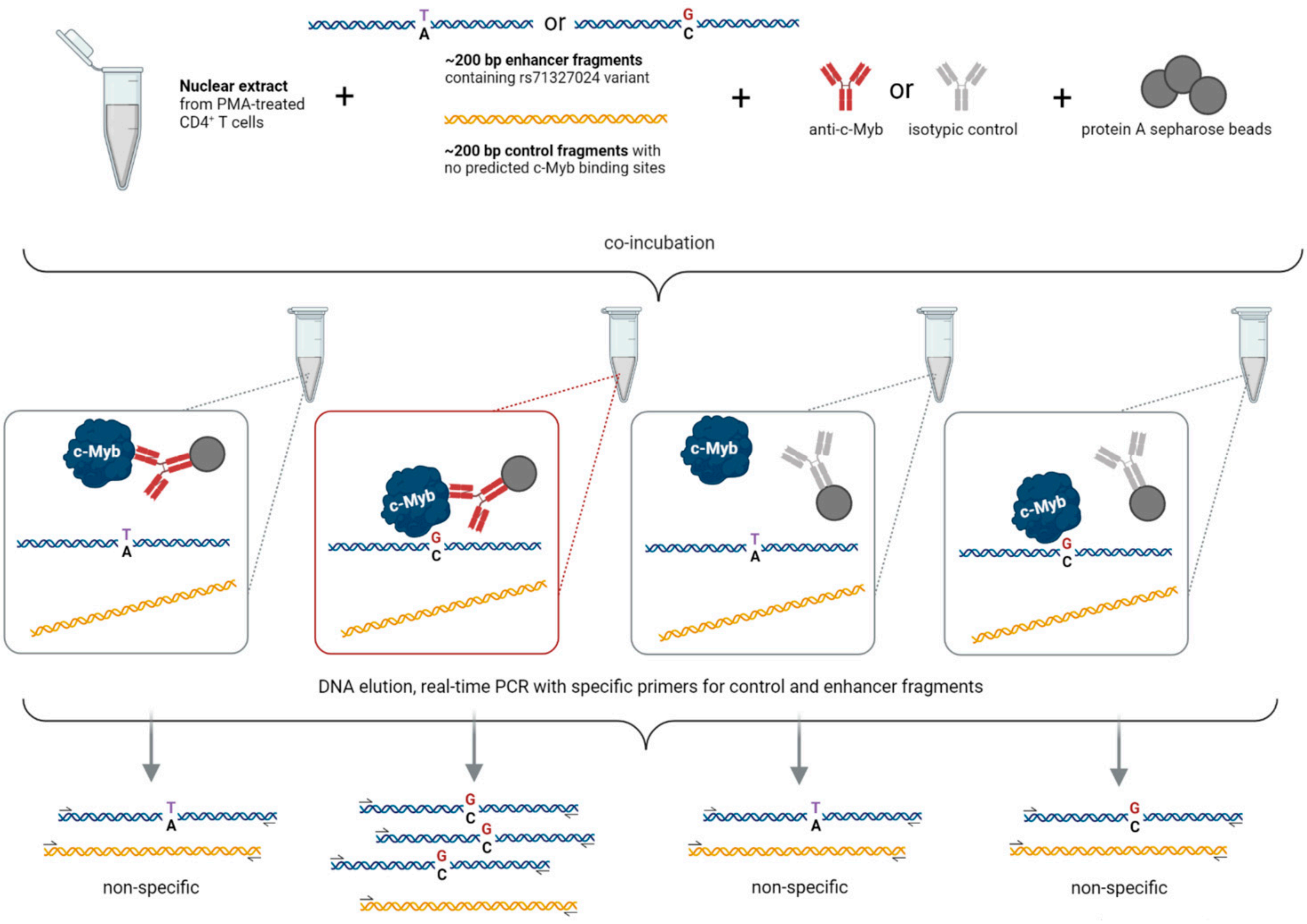

Figure S3. Scheme of the DNA pull-down assay used in the study (created with BioRender.com).

Table S1. rs71327024(T) minor allele frequencies for different populations (NCBI dbSNP database).

Minor allele frequencies	Global	American	African	East Asian	European	South Asian
**1000Genomes_30x**	0.1087	0.061	0.0034	0.0462	0.1161	0.3569
**gnomAD**	0.081548	0.06780	0.01863	0.0441	0.11609	no data
**ALFA**	0.10439	no data	0.0187	0.06	0.12537	0.32

Table S2. Oligonucleotides utilized in the study.

Primer	Nucleotide sequence 5′- 3′
prom1CXCR6-KpnI-Fw	TTAAGGTACCAAAGGCCTGCAAACCTACTAGAG
prom1CXCR6-XhoI-Rev	TTAACTCGAGGGTGTCTGTTCTGATGAACACCT
prom2CXCR6-KpnI-Fw	TTAAGGTACCAAGTGCTCAATTCAAGTGGCACTA
prom2CXCR6-XhoI-Rev	TTAACTCGAGAGGATGAGCTGAGAGATCATCTG
3p21.31-enh-BamHI-Fw	TTTTGGATCCTTCAGTTTAGAATCTGGAACTGACC
3p21.31-enh-SalI-Rev	TTTTGTCGACGTGGCTCAACATTTACATTGCAGG
contr-nonenh-BamHI-Fw	TTTTGGATCCCACATTAATGCCCTTGGGATAGTC
contr-nonenh-SalI-Rev	TTTTGTCGACCAAGGGCAATAGTGACTGGTTCC
real-time_c-Myb-Fw	ATCTCCCGAATCGAACAGATGT
real-time_c-Myb-Rev	TGCTTGGCAATAACAGACCAAC
real-time_CXCR6-Fw	CAGTTCAGCAAGGTCTTTCTGCC
real-time_CXCR6-Rev	AGGTTCACCAGGAACACATCCG
siRNA-323_c-Myb-sense	GAAAUACGGUCCGAAACGUdTdT
siRNA-323_c-Myb-antisense	ACGUUUCGGACCGUAUUUCdAdG
scrambled_c-Myb-sense	GCAUACCACGGAGUUAGAAdTdT
scrambled_c-Myb-antisense	UUCUAACUCCGUGGUAUGCdAdG
pull-down-Fw	TGGGTTCCTGGTGGTGAATG
pull-down-Rev	TGCTCCAGCCCTTGAAGAAG
pull-down-control-Fw	AGCCATGGCCTTCTGTTTT
pull-down-control-Rev	GTGTACAAGACCTGGGTGGA
overlap_rs71327024(T)-Fw	GATGTGGCATTTTTTTGCTGTGGTTATGA
overlap_rs71327024(T)-Rev	GCAAAAAAATGCCACATCAAACTGAGA
